# Phosphorus fractionation distribution in Guapimirim estuary: SE Brazil

**DOI:** 10.1186/s40064-016-3065-9

**Published:** 2016-08-24

**Authors:** Michel Arthur Faria Vicente, Gustavo Vaz de Melo, José Antonio Baptista Neto, Allan Sandes de Oliveira

**Affiliations:** Departamento de Geologia e Geofísica Marinha - LAGEMAR, Instituto de Geociências, Universidade Federal Fluminense, Av. General Milton Tavares de Souza, s/n. Campus da Praia Vermelha – Gragoatá, Niterói, RJ Brazil

**Keywords:** Phosphorus fractions, Estuary, Guapimirim, Guanabara bay

## Abstract

The Guapimirim estuary is the main tributary of Guanabara bay and is located in the northeast portion. Although it is protected, this estuary has been experiencing strong anthropogenic pressure, which has led to changes in the natural characteristics. Large amounts of sewage are dumped into the bay through tributaries, thereby changing the water and bottom sediment quality. One of the main elements of sewage is phosphorus. Despite its importance to life, a high concentration of this nutrient in the environment can result in eutrophication. This work describes the phosphorus distribution in its different fractions in the bottom sediment at 16 stations located in the main channel of the Guapimirim estuary. These results are correlated with data on grain size, organic matter and calcium carbonate content in the bottom sediment and with physicochemical parameters of the bottom water. The grain size decreases toward the mouth of the estuary, whereas the organic matter and carbonate content increase. The salinity increases significantly at 3.5 km upstream from the mouth, where there is also a notable increase in fine sediments; the same site is the mean position of the salinity front. The temperature and pH increase in the same direction. The P_inorg_-total ranges between 3.18 and 7.13 µmol g^−1^, increasing toward the mouth. The same trend is observed for the other phosphorus fractions P-Ca, P-Fe and P-f.a., which range from 0.68 to 1.91, 0.79 to 1.71 and 0.03 to 0.93 µmol g^−1^, respectively. The P-Ca and P-Fe fractions are the most representative in the P_inorg_-total, occurring at 26.3 and 26.0 %, respectively.

## Background

Estuaries are complex environments where strong interactions occur between the rivers and the sea (Chen et al. 2012). These transitional environments are often characterized by strong gradients, high variability of abiotic factors and high biological productivity (Whitfield et al. 2013; Basset et al. 2013). Therefore, estuaries are considered one of the most productive ecosystems in that they serve as a nursery for several species and playing an important role in sustaining coastal fisheries and support recreational and cultural activities (Ortega-Cisneros et al. 2014). These characteristics make estuarine regions very favorable to human occupation, which often leads to a dense population and the economic development of these areas. Despite their importance, estuaries around the world have been experiencing drastic changes in water and sediment quality (Chen et al. 2012). Due to the rapid urban growth of these areas, sewage has become one of the main causes of pollution (Teodoro et al. 2010) and directly impacts the trophic levels of estuaries.

The Guapimirim estuary, which is located in the northeastern portion of Guanabara bay at Rio de Janeiro, Brazil, has experienced such changes. Over the last 100 years, the catchment area around this bay has been strongly modified by human activities, which have increased river discharge, sediment loads and the amount of waste and untreated sewage entering the bay (Borges et al. 2009; Aguiar et al. 2011; Fonseca et al. 2013; Baptista Neto et al. 2013). As the largest contributor of fresh water to Guanabara bay, the Guapimirim estuary also brings in nutrients, especially phosphorus (P), from sewage and various human activities in its catchment area. Phosphorus is an essential nutrient that plays a key role in biogeochemical cycles of coastal environments (Meng et al. 2014a). However, its excessive input caused by human activities can lead to eutrophication, which negatively affects coastal environments around the world (Statham 2012; Shao et al. 2013; Jickells et al. 2014; Kraal et al. 2015). It not only degrades water quality but also causes other diverse problems, such as harmful algal blooms that may pose a serious health hazard to humans (Chen and Yu 2011).

To better understand phosphorus dynamics in estuaries, this research investigated the phosphorus fraction distribution associated with bottom sediments along the main channel of the Guapimirim estuary. The survey also associated each fraction with grain size characteristics, organic matter concentrations, calcium carbonate and physicochemical parameters of bottom water (temperature, salinity, dissolved oxygen, pH and Eh). The data were compared with the salinity front (SF), and a conceptual model of phosphorus fraction distribution along the salinity gradient in Brazilian southeast estuaries was proposed.

## Methods

### Study site

**Fig. 1:**
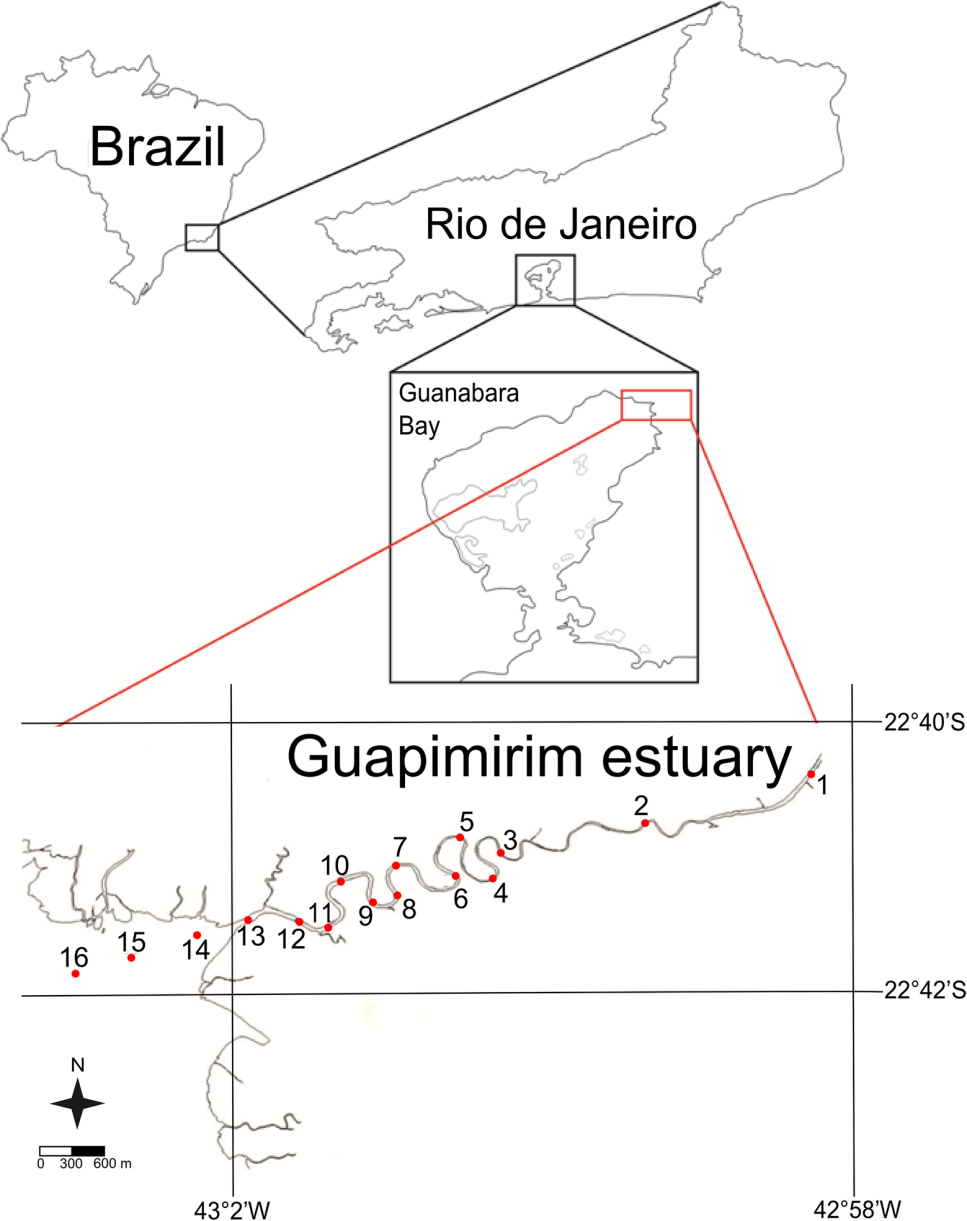
Map of Guapimirim estuary inside the Guanabara Bay, Rio de Janeiro state, showing the sampling stations

The Guapimirim estuary is located in the northeast section of Guanabara bay, at 22°40′–22°42′S, 42°58′–43°03′W, within the Guapimirim Environmental Protection Area (APA) (Fig. 1). This protected area was established on September 25, 1984, by Federal Decree 90225, to preserve the remaining mangroves within Guanabara bay. The Guapimirim estuary is the main tributary of this bay, contributing approximately 20.8 % of the total volume of incoming freshwater (Kjerfve et al. 1997). The largest tributaries of the research estuary are the Macacu, Guapiaçú and Guapimirim rivers. The catchment area extends over 1.640 km^2^ and is bounded to the north and northwest by the Serra dos Órgãos, to the northeast by the Serra de Macaé de Cima, to the east by the Serra da Botija e Monte Azul and to the south by the Serra do Sambe and Garcias. Before discharging into Guanabara bay, the Guapimirim river meanders across an extensive mangrove area. The depth of its main channel is approximately 3 m but reaches 5 m in some places. This estuary experiences micro and mixing tides; the amplitude of the neap tide is approximately 40 cm, whereas the spring tide can reach approximately 150 cm (Melo et al. 2014).

The catchment area of the Guapimirim estuary is very important for the supply of cities such as Cachoeira de Macacu, Guapimirim, São Gonçalo, Itaboraí and Niterói. To meet the region’s demand, this catchment area was altered by the former Departamento Nacional de Obras e Saneamento (DNOS) in the 1950s. The main change was the creation of the Canal de Imunana to collect water for the Laranjal treatment plant in São Gonçalo. This work diverted the course of the Macacu river, connecting it to the Guapimirim river and increasing the catchment area of the Guapimirim estuary. The region near the mouth of the studied estuary retained its original features.

The land use in the estuary’s catchment area, which is predominantly rural with native vegetation and few urban centers, has changed in recent years. The region has experienced socio-spatial transformations and population growth, which were generated by the deployment of a large petrochemical complex (COMPERJ) and other activities related to the oil and gas sector (Klink 2013; Baptista Neto et al. 2013).

### Field surveys and analysis methods

The survey was performed in July 2010. The sediment samples were collected along the main channel of the Guapimirim estuary at sixteen stations that were approximately 300 m apart. The samples were collected using a stainless steel Van Veen sampler. We also checked the physicochemical parameters of the bottom water (temperature, salinity, pH, Eh and dissolved oxygen) in the sediment sampling locations using a CTD SBE 19 PLUS—Seabird sensor. The sample stations are shown in Fig. 1.

After surveying, the sediments were cooled and taken for analysis to the sedimentology laboratory of the Universidade Federal Fluminense, where they were freeze-dried. The samples were divided into two subsamples each. The first subsamples were used to determine the organic matter content by difference after ignition (Bale and Kenny 2005), to eliminate carbonates by acidification in 10 % HCl (Schubert and Nielsen 2000) and to analyze grain size using the particle size analyzer Malvern Mastersizer 2000 (Malvern). The sediments were classified according the textural classification of Flemming (2000).

The other subsamples were subjected to phosphorus fractionation according to Brepohl (2000), which was adapted from Psenner et al. (1988). This method allows the extraction of the following phosphorus fractions in sediment: Weakly adsorbed phosphorus (P-f.a.), Phosphorus associated with manganic and ferric colloids (P-Fe), Phosphorus bound to metal oxide (P-Me), Carbonates bound to phosphorus (P-Ca) and residual phosphorus (P-res). Subsequently, the extracts were analyzed using a Perkin Elmer UV/Vis Lambda-12 molecular absorption spectrophotometer. The analytical data quality was guaranteed through the implementation of quality control methods, including calibration, analysis of reagent blanks and analysis of replicates. The Pearson correlation was used to verify the relationship level for all analyzed data.

## Results and discussion

The quantification of total phosphorus concentrations is considered a key factor for determining the eutrophication levels in water ecosystems. However, the knowledge of total phosphorus concentrations alone is not sufficient to determine the risk associated with its presence in natural water bodies (Gaspar et al. 2013). The lack of phosphorus fractionation data in estuarine sediments of Guanabara bay is surprising. There are few studies regarding the inorganic fractions of sedimentary phosphorus in estuarine systems of this important Brazilian bay (Rangel et al. 2013). The study of the transformation dynamics of P fractions is important for a better understanding of the availability of different P fractions in these environments (Yang et al. 2012). The results found in the present research help remedy this lack of information in the Guapimirim estuary.

The spatial variability of particle size characteristics of Guapimirim estuary sediments results from a complex interaction between the hydrodynamics and geomorphology. The main channel of the Guapimirim estuary contained *Sand* and *Muddy Sand*. The mud percentage (silt + clay) ranged from 0.7 % at station 2 to 46.69 % at station 14. The grain size distribution along the estuary shows finer sediments from station 9 toward station 16, which is located at the estuary mouth, where a large amount of *Muddy Sand* occurs. The highest occurrence of *Sand* is found in the upper estuary, which reflects greater hydrodynamic energy. In contrast, the *Muddy Sand* is related to weaker hydrodynamic conditions (Flemming 2000), indicating lower current energy in this sector of the estuary. Studies examining saline intrusion dynamics in response to the physical processes of river flow and tides indicate the existence of a SF in the region near the mouth of Guapimirim estuary (Melo et al. 2014), where the interface between these physical processes could favor the deposition of fine sediments. In this estuary sector, processes such as flocculation occur more intensely due to the reduced turbulence, which favors the finer sedimentation (Geyer et al. 2004). Other studies also show that the grain size is regulated by local hydrodynamics. In these studies, grain size is related to phosphorus affinity. Sectors with higher hydrodynamic conditions are often dominated by larger grain sizes and generally lower levels of sedimentary phosphorus. In contrast, sectors with lower hydrodynamic conditions have smaller grain sizes, with a higher proportion of silt and clay, which tend to adsorb more phosphorus compounds due to the large surface contact area (Zhu et al. 2012; Cotovicz Junior et al. 2014; Gao et al. 2014; Tang et al. 2014).

We verified an increase in fine sediments and a decrease in sand toward the estuary mouth. The organic matter content also increased in the same direction. This percentage ranged from 1.08 % at station 2 to 13.48 % at station 15. The same increasing tendency was observed in calcium carbonate content, which ranged from 0.80 % at station 4 to 9.75 % at station 15. Calcium carbonate is produced by marine organisms in the form of two main polymorphs, calcite and aragonite. It is very important to verify the carbonate content in sediments because carbonate can immobilize phosphorus. Carbonate solubility is mainly affected by the pressure, temperature and pH (Hauck et al. 2012; Zaaboub et al. 2014; Mahmood et al. 2015). The bottom water salinity ranged from 0.03 to 13.48 at stations 1 and 15, respectively. From station 1 to station 9, approximately 3.5 km upstream from the mouth, the salinity was approximately 0, showing the strong fluvial influence in this sector. At the downstream stations, salinity increased significantly. Bottom water temperature ranged from 20.89 °C at station 1 to 22.93 °C at station 14, following the salinity variation. In a study on sedimentary phosphorus dynamics in urbanized and non-urbanized rivers in southern Brazil, the authors found a similar tendency of increasing organic matter, calcium carbonate content and smaller grain size toward the sea (Pagliosa et al. 2005). Another study also found a significant positive correlation among organic matter, carbonates, fine sediment and the analyzed phosphorus forms (Gaspar et al. 2013). The results found in the Guapimirim estuary agree with the data from the cited literature. The geographical positions, grain size data, organic matter content, calcium carbonate and bottom water physicochemical parameters are shown in Table 1 for each station sampled.

**Table 1:** Sampling stations coordinates, sand, silt, clay, mud, OM and CaCO_3_ percentages and bottom water physicochemical characteristics of Guapimirim Estuary

Station	Coordinates		Sand	Silt	Clay	Mud	OM	CaCO_3_	Temperature	Salinity	pH	Eh	DO
	lat	long	(%)	(%)	(%)	(%) silt + Clay	(%)	(%)	(°C)				(mg L^-1^)
1	−*22.*6770	−42.9741	98.13	1.87	0.00	1.87	2.35	0.01	20.89	0.03	6.91	260.04	6.33
2	−22.6823	−42.9911	99.22	0.78	0.00	0.78	1.08	1.26	21.08	0.03	6.95	316.17	6.66
3	−22.6855	−43.0057	97.64	2.36	0.00	2.36	1.65	1.17	21.13	0.03	6.91	328.90	6.41
4	−22.6883	−43.0065	97.40	2.60	0.00	2.60	1.91	0.80	21.12	0.04	6.91	339.86	6.38
5	−22.6838	−43.0098	98.18	1.82	0.00	1.82	1.42	0.90	21.13	0.04	6.93	335.00	6.40
6	−22.6879	−43.0103	95.73	4.14	0.13	4.27	2.45	1.98	21.16	0.07	6.94	332.81	6.41
7	−22.6868	−43.0164	91.49	8.00	0.52	8.52	1.86	2.11	21.25	0.16	6.95	320.77	6.16
8	−22.6900	−43.0161	76.36	22.48	1.16	23.64	4.73	2.96	21.31	0.24	6.99	316.35	6.08
9	−22.6908	−43.0187	91.19	8.47	0.34	8.81	3.51	1.64	21.68	1.54	7.00	318.94	5.87
10	−22.6884	−43.0220	58.67	39.57	1.76	41.33	9.28	5.45	22.52	2.15	7.14	306.54	6.08
11	−22.6935	−43.0233	89.57	10.06	0.37	10.43	1.85	2.76	22.83	4.49	7.32	299.06	6.33
12	−22.6928	−43.0264	77.33	21.97	0.70	22.67	4.93	4.41	22.87	8.87	7.42	301.64	6.19
13	−22.6927	−43.0314	81.37	18.06	0.57	18.63	6.42	5.53	22.79	8.89	7.52	299.26	3.60
14	−22.6944	−43.0366	53.31	45.00	1.69	46.69	10.30	6.69	22.93	13.14	7.75	290.33	6.54
15	−22.6968	−43.0433	54.20	44.49	1.31	45.80	13.48	9.75	22.81	13.53	7.76	279.19	6.70
16	−22.6986	−43.0489	66.12	32.49	1.39	33.88	8.10	6.39	22.75	13.32	7.79	271.81	6.70

**Fig. 2:**
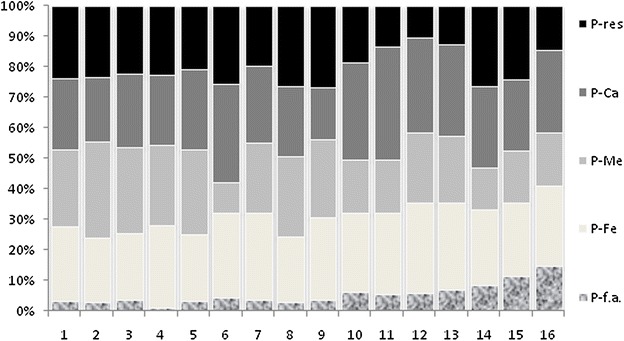
Relative percentage of phosphorous fraction in sampling stations

**Fig. 3:**
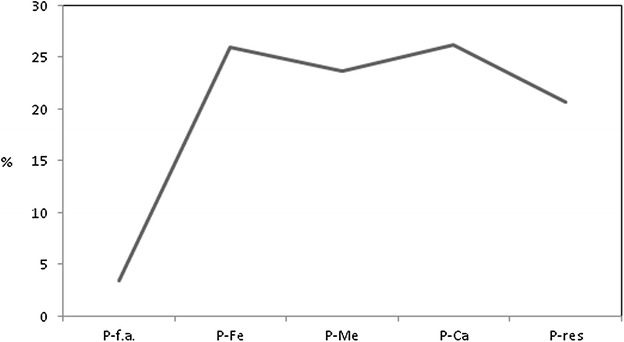
Phosphorous fractions medium percentage

Table 2 presents the P fractionation data for each sampled station. P-f.a. showed an increase in concentration from the upper sector toward the estuary mouth. This fraction presented concentrations that ranged from 0.03 µmol g^−1^ (station 4) to 0.93 µmol g^−1^ (station 16), with a mean value of 0.29 µmol g^−1^. This fraction behaved similarly to organic matter, carbonate content and fine grain size. According to studies on phosphorus fractionation (Brepohl 2000), sedimentary organic matter contributes to the retention of phosphorus by the sediment. Thus, the higher the concentration of sedimentary organic matter is, the higher the concentration of this fraction is, as found in this study. This relationship was verified and corroborated by the significant positive correlation of this fraction with sedimentary organic matter (*p* = 0.83). Particle size is also an important factor in controlling the content of P-f.a. in sediments. Sediments with smaller particle sizes have stronger adsorption capacities due to their greater specific surface areas (Zhuang et al. 2014). This was verified by the positive correlation between P-f.a. and silt (*p* = 0.80) and clay (*p* = 0.72). A research study in the Yangtze estuary and Hangzhou bay (Li et al. 2013) similarly found the largest storage capacity of phosphate to occur in fine particles. The bioavailability and mobility of phosphorus in sediments depend on the original existing chemical forms (Wang et al. 2013), and alterations in physical and chemical factors, such as temperature, pH, water dynamic conditions, bioturbation and the redox characteristics, could lead to the release of P-f.a. into the water column (Chen et al. 2011). Figure 2 shows the relative percentage of phosphorus fraction in the sampled stations. P-f.a. was measured at an average of 3.4 % of the total inorganic phosphorus (P_inorg_-total) content, as shown in Fig. 3.

**Table 2:** Phosphorus fractionation results in Guapimirim Estuary (μmol g^−1^)

Station	P-f.a.	P-Fe	P-Me	P-Ca	P-res	P_inorg_-total
1	0.12	0.92	0.93	0.88	0.89	3.73
2	0.13	0.90	1.34	0.89	1.00	4.25
3	0.13	0.79	1.02	0.87	0.81	3.62
4	0.03	1.00	0.97	0.85	0.83	3.69
5	0.14	0.98	1.23	1.17	0.92	4.45
6	0.14	0.89	0.31	1.03	0.81	3.18
7	0.15	1.15	0.93	1.02	0.79	4.03
8	0.13	1.04	1.26	1.09	1.27	4.80
9	0.15	1.09	1.02	0.68	1.08	4.02
10	0.35	1.41	0.97	1.77	1.02	5.52
11	0.29	1.37	0.90	1.91	0.70	5.17
12	0.30	1.50	1.17	1.56	0.54	5.06
13	0.37	1.50	1.13	1.57	0.66	5.23
14	0.53	1.56	0.86	1.69	1.66	6.30
15	0.82	1.71	1.20	1.68	1.72	7.13
16	0.93	1.65	1.08	1.71	0.90	6.27

P-Fe is an important indicator of phosphorus mobility. This fraction is significantly affected by oxygen and pH (Hou et al. 2008; Ren et al. 2012; Zhang et al. 2015) and is often used as an indicator of sediment quality and environmental pollution levels (Xiang and Zhou 2011; Yuan et al. 2013). P-Fe is formed by the co-precipitation of phosphate with Fe oxides and hydroxides (Meng et al. 2014a). This fraction is considered an easily resolved constituent of sediments because it may change with redox changes in the environment. When the redox potential (Eh) is lowered, the number of iron ions is reduced, P is released, and P concentrations in the water may increase. When Eh increases, active iron is oxidized and rapidly adsorbs P from the water column, and the P concentrations in water may decrease (Zhuang et al. 2014). The Eh measured 260 at station 1 and 316 and 328 in the following stations. At station 16, Eh reached 271. In the Guapimirim estuary, P-Fe showed an increasing trend from the upper estuary to its mouth; however, the concentration was higher than the previous fraction. This fraction presented concentrations that ranged from 0.79 µmol g^−1^ (station 3) to 1.71 µmol g^−1^ (station 15). This was the second most representative fraction in P_inorg_-total average content, at 26 % (Fig. 3). There were no significant changes in this fraction along the estuary because there were no major changes in oxygen and pH conditions. The dissolved oxygen in the bottom water showed concentrations ranging from 5.87 mg L^−1^ (station 9) to 6.70 mg L^−1^ (station 16), thus revealing good oxygenation along the main channel of the Guapimirim estuary. The pH values ranged from 6.9 to 7.8, which was within the range expected for estuarine regions, which reduces nutrient availability from sediments to the environment (Jin et al. 2006). P-Fe was also positively correlated with fine sediments (silt and clay, *p* = 0.85 and *p* = 0.78, respectively) and with P-f.a. (*p* = 0.86). The Pearson correlation for each variable is shown in Table 3. Other studies also found a significant correlation between the P-f.a. and P-Fe, thus indicating that P-Fe is an important phosphorus source (Yuan et al. 2013).

**Table 3:** Pearson correlation matrix for the phosphorous fraction and others physicochemical characteristics of Guapimirim Estuary

	P-f.a.	P-Fe	P-Me	P-Ca	P-res	P_inorg_-total	%sand	%silt	%clay	%OM	%CaCO_3_	Salinity	Temperature	pH	Eh	DO
P-f.a.	1.00															
P-Fe	0.86	1.00														
P-Me	0.14	0.17	1.00													
P-Ca	0.72	0.86	0.05	1.00												
P-res	0.44	0.31	0.14	0.15	1.00											
P_inorg_-total	0.89	0.92	0.35	0.83	0.57	1.00										
%sand	−0.80	−0.85	−0.12	−0.74	−0.63	−0.90	1.00									
%silt	0.80	0.85	0.12	0.74	0.63	0.90	−1.00	1.00								
%clay	0.72	0.78	0.10	0.70	0.57	0.82	−0.97	0.96	1.00							
%OM	0.83	0.83	0.12	0.67	0.67	0.89	−0.96	0.96	0.88	1.00						
%CaCO_3_	0.89	0.91	0.14	0.78	0.54	0.93	−0.93	0.93	0.83	0.15	1.00					
Salinity	0.90	0.92	0.15	0.75	0.39	0.88	−0.79	0.79	0.67	0.81	0.89	1.00				
Temperature	0.76	0.94	0.10	0.90	0.19	0.84	−0.78	0.78	0.70	0.74	0.84	0.87	1.00			
pH	0.92	0.94	0.13	0.80	0.38	0.90	−0.81	0.81	0.70	0.81	0.90	0.99	0.90	1.00		
Eh	−0.06	−0.60	−0.13	−0.50	−0.27	−0.61	0.52	−0.52	−0.46	−0.56	−0.52	−0.64	−0.52	−0.64	1.00	
DO	0.54	0.29	0.10	0.31	0.20	0.40	−0.18	0.18	0.04	0.29	0.40	0.52	0.23	0.52	−0.33	1.00

As seen in the results, the P-Me fraction did not show regular behavior. This fraction was not significantly correlated with any of the other parameters. Because of this irregular distribution along the estuary, it was not possible to verify the parameters of influence on its content. P-Me was the third most represented fraction in P_inorg_-total content, as shown in Fig. 3.

**Fig. 4:**
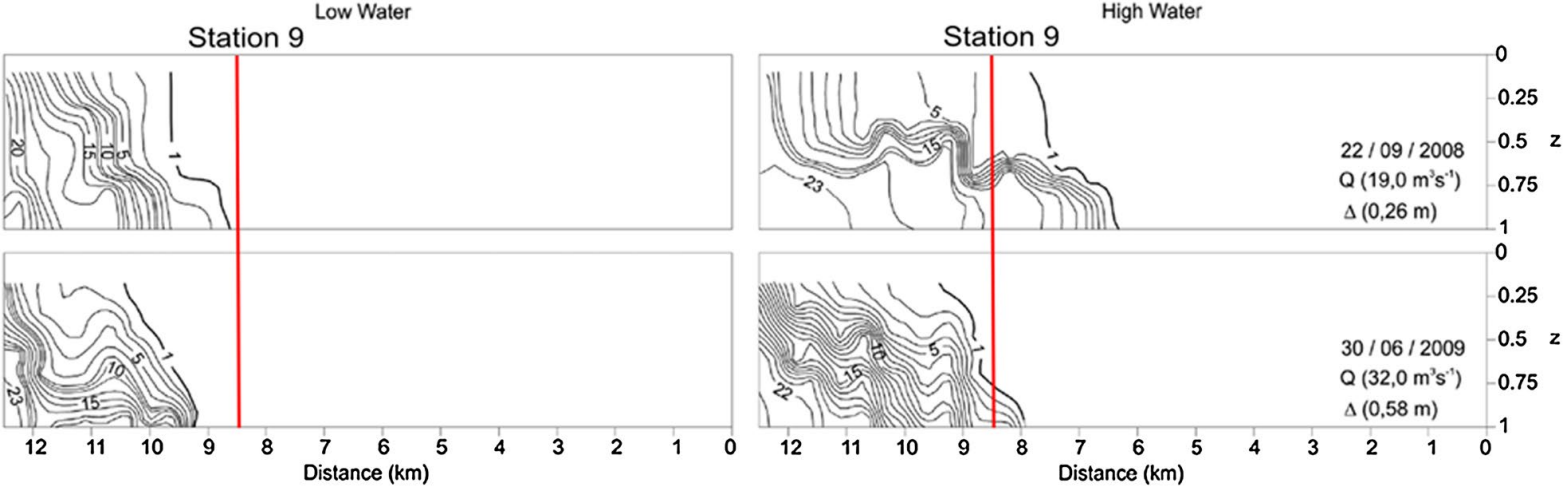
Longitudinal variation of salinity in the Guapimirim estuary, during dry season in neap tide. Q = freshwater discharge and ∆ = tidal range during sampling (Adapted from Melo et al. 2014)

P-Ca is mainly originated from the detritus of biological bones, P combined with calcium carbonate and carbonate fluorapatite, which can precipitate in interstitial water (Zhuang et al. 2014). In the Guapimirim estuary, P-Ca presented the same behavior as did the P-f.a. and P-Fe fractions, increasing from the upper sector (station 1) toward the estuary mouth (station 16). There was a significant increase from station 9, which is located 3.5 km upstream from the mouth. At this station, the salinity increased substantially. This station can be identified as the midpoint of the salinity front (Fig. 4). In freshwater systems, salinity changes affect a suite of biogeochemical processes. An increase in salinity leads to increased Ca^2+^ concentrations, which may result in the immobilization of P by co-precipitation with Ca^2+^ in calcium carbonate (van Diggelen et al. 2014). The three fractions described thus far (P-f.a., P-Fe and P-Ca) tended to be abundant in smaller sediment grain sizes because their larger surface areas can increase the adsorption of phosphates, metals and organic matter (Meng et al. 2014b). Although P-Ca was more stable than P-Fe, this fraction was significantly regulated by pH and the carbonate content (Ren et al. 2012; Wang 2012; Gireeshkumar et al. 2013; Zhang et al. 2015). A study on the fractionation and bioavailability of phosphorus in a tropical estuary in southwestern India indicated that high concentrations of P-Ca might be interpreted as the result of the accumulation of calcium in estuarine environments under favorable higher salinity and pH conditions (Renjith et al. 2011). The same study indicated that salinity controls the flocculation and sedimentation mechanisms in estuarine environments. P-Ca was the main P pool of this Indian estuary. The researchers also identified this fraction as an important and potentially dominant phase of P in shallow-water tropical carbonate-rich sediments. Another study also found P-Ca as the most representative fraction in the analyzed marine sediments in Kalpakkam, India (Bramha et al. 2014). The Guapimirim estuary results also indicated that this fraction was the most representative in the P_inorg_-total content, at 26.3 %. In addition, this fraction showed a significant positive correlation with silt (*p* = 0.74), CaCO_3_ (*p* = 0.78), salinity (*p* = 0.75), and higher pH (*p* = 0.80).

As seen in the results, P-res did not show significant variations along the estuary. The P-res concentrations ranged from 0.54 µmol g^−1^ at station 12 to 1.72 µmol g^−1^ at station 15; the second-highest concentration was found at station 14, with 1.66 µmol g^−1^. The mean concentration found for this fraction was 0.98 µmol g^−1^. According to Brepohl (2000), this fraction is one of the natural characteristics of the sediment. The two highest concentrations observed along the estuary coincide with the points of greater deposition of fine sediments, indicating that the hydrodynamic conditions can influence in its redistribution along the estuary.

**Fig. 5:**
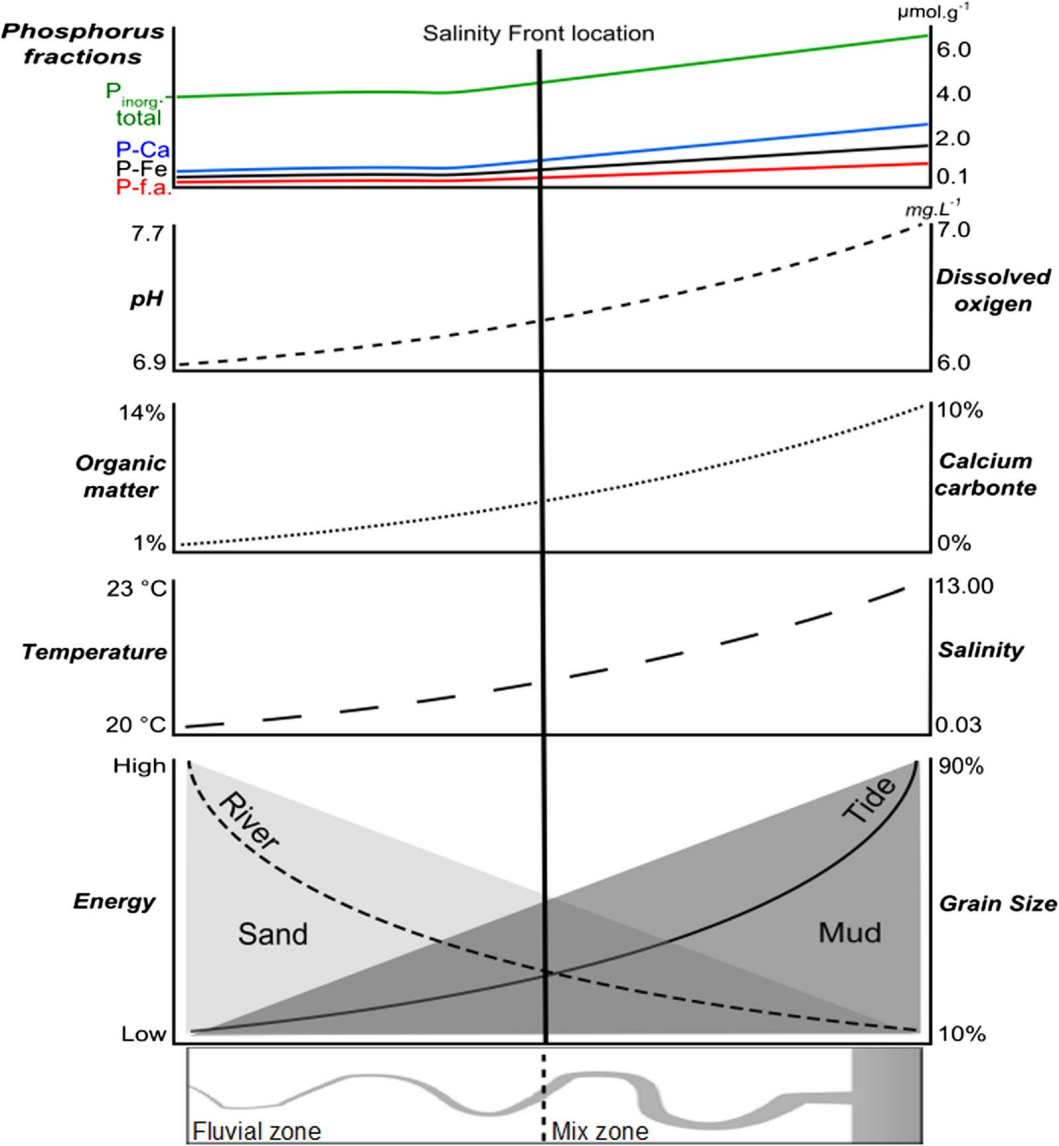
Conceptual model of phosphorous fractions distribution along the salinity gradie in Brazilian southeast estuaries

The estuary sediments may act as a P source or sink, which is very important in regulating the trophic status of the overlying water and/or ambient pore water (Zhang et al. 2012; Jin et al. 2013). The sum of the five fractions analyzed in this work corresponds to the total inorganic phosphorus (P_inorg_-total). In the Guapimirim estuary, P_inorg_-total ranged from 3.18 to 7.13 µmol g^−1^. These results are within the average range from 0.84 to 20.58 µmol g^−1^ found in the Cananéia-Iguape estuarine system in SE Brazil (Barcellos et al. 2005). According to the authors, these results are compatible with other estuaries around the world. In the Guapimirim estuary, the higher P_inorg_-total concentrations were associated with lower hydrodynamic conditions and with finer sedimentation, as also found in several other studies (Barcellos et al. 2005; Zhu et al. 2012; Li et al. 2013; Berbel and Braga 2014; Cotovicz Junior et al. 2014; Zhuang et al. 2014). The differences in the percentages of each fraction observed in the various regions around the world are due to the existing environmental features in each coastal system, such as the types of impacts on the aquatic ecosystems. Figure 5 proposes a conceptual model of phosphorus fraction distribution along the salinity gradient in Brazilian southeast estuaries. This model considers the river and mixing sectors as well as variations of the physicochemical characteristics of bottom water and particle size. The environmental hydrodynamic conditions are reported, and the average limit of the SF is highlighted.

## Conclusion

Estuaries are complex environments due the interaction between terrestrial and aquatic processes. The river flow and tidal force are two important energy sources that create areas with different characteristics along the estuary. The locations of zones created by this interaction depend on flow and tide conditions. In the estuarine mixing zone, there is a very peculiar feature called the salinity front (SF). In the SF zone, actuation causes a decrease in hydrodynamic conditions that are generated by the reciprocal annulment trend of energy involved in this process. Significant changes in water and sediment characteristics also occur in this area. In the Guapimirim estuary, the upper zone had predominantly sandy sediments due to the higher capacity of river transport. The mixing zone had more fine sediments, organic matter and calcium carbonate content due by the lower hydrodynamic conditions. Starting from that zone and moving toward the marine zone, an increase in temperature, salinity, dissolved oxygen and pH of bottom water occurred. The results found in the present study showed that the greatest P-f.a. concentrations were verified in the estuarine mixing zone and were associated with finer grain size, greater organic matter content and a decrease in hydrodynamic conditions. These factors also influenced the highest concentrations of P-Fe in the mixing zone, in addition to bottom water oxygenation. P-Me showed no regular distribution along the estuary. P-Ca also had greater concentrations in the mixing zone; however, their concentrations were higher than the first two fractions due to the alkalinity of the bottom water in the mixing zone. This characteristic impeded the redissolution of phosphorus immobilized by the high carbonate availability in this zone. P-res showed no regular distribution along the estuary. This fraction showed peak concentrations only at lower hydrodynamic points, which indicates that these conditions can influence the redistribution of this fraction. P_inorg_-total had higher concentrations in the mixing zone. This work suggests that the limit between the mixing zone and the river zone coincides with the SF position. In the Guapimirim estuary, this limit is located at station 9, approximately 3.5 km upstream of the mouth, on average.
